# Understanding the pathways between the use of urban green spaces and self-rated health: A case study in Mexico City

**DOI:** 10.1371/journal.pone.0295013

**Published:** 2023-12-07

**Authors:** Carolina Mayen Huerta

**Affiliations:** School of Architecture and Landscape Architecture, University of Edinburgh, Edinburgh, United Kingdom; University of Turin: Universita degli Studi di Torino, ITALY

## Abstract

In recent decades, extensive research has demonstrated the positive impact of urban green spaces (UGS) on public health through several pathways. However, in the context of Latin America, particularly Mexico City, there remains a notable scarcity of evidence linking UGS use to health outcomes and an insufficient understanding of the pathways or factors underlying these associations. Therefore, this study employs Structural Equation Modeling (SEM) to investigate the intricate pathways between UGS use and residents’ perceived health in Mexico City, a densely populated urban center. The SEM integrates three key mediators: sentiments towards UGS, UGS quality, and time spent within these spaces. Survey data was collected through an online survey distributed via social media in May 2020 (n = 1,707). The findings indicate a minor yet significant direct link between UGS use and self-reported health (0.0427, *p* < 0.1). Conversely, the indirect pathways through sentiments towards UGS, UGS quality, and time spent in UGS were highly significant (0.1950, *p* < 0.01), underscoring their substantial role as mediators in the UGS use-health association. While a comprehensive understanding of the mechanisms linking perceived health to UGS use in Mexico City requires further research, this study proposes that fostering positive sentiments towards UGS, enhancing UGS quality, and encouraging extended visits to green areas could potentially amplify the perceived health benefits associated with UGS use among residents. These insights offer valuable inputs for policymaking, emphasizing the importance of integrating public perspectives to optimize nature-based solutions and broaden their positive impact within Mexico City.

## 1. Introduction

Since the early 19th century, researchers have increasingly recognized the beneficial effects of nature on human health and well-being [1, 2]. This acknowledgment has grown in recent decades, with green spaces’ positive impact progressively underscored in the public health literature [3–8]. From public parks and woodlands to gardens in hospitals and care homes, individuals and institutions have used green spaces recreationally and formally to improve physical and mental health outcomes, such as stress reduction, mood improvements, and lower rates of cardiovascular disease [9, 10]. In particular, in fast-growing cities, urban green spaces (UGS) are deemed an effective policy tool to reduce health inequalities, promoting well-being and a better quality of life, and thus counteracting some of the negative impacts of rapid urbanization [11–14].

However, specific evidence on the mechanisms linking green space use to improvements in health outcomes in Latin American megacities is lacking, and only a few studies have examined in-depth the pathways or factors mediating some type of relationship between green spaces and health or well-being and evidence has not been conclusive in all cases [15]. For instance, Bonilla-Bedoya et al. (2020) [16] delved into the pathways connecting urban green areas and perceived restoration in Quito. However, their study did not find conclusive evidence linking green spaces to subjective well-being. In a similar vein, Ordóñez Barona et al. (2023) [17] investigated the pathways involving collectively made decisions on green spaces and satisfaction, a well-being indicator, across 10 Latin American cities. They discovered significant indirect pathways involving contact with green spaces, trust, and effective management. Finally, Aldana-Domínguez et al. (2022) [18] explored the contributions the contributions of distinct ecosystem types to human well-being in Barranquilla, Colombia. The authors established a significant and positive connection between ecosystem services and well-being, particularly through factors such as freedom of action, access to basic materials, and social relationships.

To date, little attention has been paid to the effect of using UGS to enhance health outcomes in the densely populated cities of Latin America, where wealth inequalities are vast, and the availability of green spaces is often scarce [19–26]. Moreover, there remains a lack of understanding or appreciation regarding UGS as health-promoting environments that can help mitigate urban inequalities, and the significance of UGS in reducing social vulnerability is often downplayed [27–29]. In addition, studies analyzing the relationship between green spaces and health rarely consider more than one mediator at a time [30, 31], and there is a lingering question regarding the transferability of findings from high-income countries to Latin American settings [32]. Although studies in high-income countries have examined various mechanisms linking green space use to health, including physical activity and air pollution, evidence supporting these hypotheses in the global South remains limited, with contextual differences possibly accounting for [33, 34].

Accordingly, the present study aims to address these gaps by examining the pathways between the use of UGS and the self-reported health of residents of Mexico City, accounting for multiple mediators through Structural Equation Modeling (SEM). In the context of hypothesis testing and theory development, this approach is suitable as it helps in understanding how variables directly and indirectly affect one another. Mexico City was chosen as a case study because it presents patterns of spatial segregation, extreme social inequality, and a dense population of 8,400 people per square kilometer [35, 36]. In recent decades, the Latin American megacity of 1,479 km^2^ has experienced accelerated growth rates, exacerbating spatial inequalities that have contributed to increasing public health risk factors [36]. Furthermore, urban planning strategies have largely ignored green spaces and their potential health benefits as an important consideration in urban improvement policies [37–39].

As such, this study will seek to determine how the use of green spaces is directly or indirectly associated with Mexico City’s residents’ perceived health through multiple mediators, namely sentiments toward green spaces, UGS quality, and time spent in UGS. The evidence generated will assist the decision-making process regarding the most effective underlying mechanisms to support health through UGS use in the city. The consideration of mediating factors is indispensable in contexts such as Mexico City, where both spatial and social inequalities may influence the perception and use of health-promoting urban environments, as well as the benefits derived from them [40]. The study findings hold the potential to guide strategies that enhance public health outcomes and improve Mexico City’s livability.

### 1.1 Pathways between UGS use and self-reported health

In this study, UGS refers to built environments supporting healthy behaviors, physical activity, recreation, social contact, and overall well-being in cities [41–44]. The category encompasses various public green areas like parks, playgrounds, gardens, reserves, sports grounds, green squares, community gardens, urban forests, and vegetated plazas, whether maintained or neglected, with unrestricted public access [37, 45–47]. Exposure to green spaces can take three forms: direct contact, indirect contact (window views), and incidental contact (transitory exposure on the way to another destination [48]. This research primarily focuses on direct contact with UGS, involving individuals’ use of these spaces and their frequency of use, which is strongly associated with improved health outcomes [49–51].

Although several studies have linked simple exposure to green spaces, which includes indirect and transitory contact, as a contributor to health [52–54], this association is not universally established, particularly in Latin American countries [55]. On the other hand, frequent UGS use is consistently linked to improved health outcomes in various contexts. For instance, the relationship between frequent UGS use and improved health has been confirmed by Coombes et al. (2010) [56] in their study of parks in Bristol, England, with participants who used UGS more frequently reporting better health outcomes. Similarly, based on data from Italy and the UK, Lafortezza et al. (2009) [57] found that frequent visits to UGS generated significant improvements in perceived well-being. In Mexico, a recent investigation found a significant relationship between the frequent use of UGS, one or more times per week, and improved health outcomes [58]. Thus, this study considers the frequency of direct UGS use as a variable of interest rather than simple UGS use or exposure.

To date, several pathways have been suggested between health and UGS use [33, 59]. Since social and spatial inequalities affect UGS perceptions and behaviors within the study area, this analysis will examine three primary mediators: sentiments toward green spaces, UGS quality, and time spent in UGS. These mediators, in turn, generate different pathways that intervene in the association between the frequency of UGS use and respondents’ self-reported health.

The first pathway examined in this study is the one mediated through sentiments toward UGS, which were incorporated into the model following Wan and Shen’s (2015) [31] framework on the mediating effects of attitudinal measures on health. The authors argue that individuals’ sentiments or attitudes predict their behavior as they influence their dispositions, including the intention to use UGS. Several authors have also contended that the perceptions acquired thanks to space can often mediate the relationship between its use and its perceived benefits since negative perceptions, feelings, and attitudes may inhibit use [55, 60–63]. Therefore, strengthening the relationship between UGS use and improved health may involve interventions aimed at changing individuals’ sentiments or attitudes, which do not suggest modifying the urban environment per se but altering the predispositions of users to maximize the benefits UGS provide [31, 64].

The second pathway between UGS use and health explored in this analysis is the one mediated through UGS quality, given its role in affecting green spaces’ type of use and enjoyment [65–69]. Multiple studies have shown that quality features influence UGS use and, therefore, the subsequent benefits individuals obtain from them [70, 71]. For example, a lack of maintenance or poor lighting in parks can lower the perception of safety, reducing the frequency of use or the time spent in such spaces [72]. Similarly, including aesthetic and visual appeal elements, which individuals associate with a space’s quality, such as flowers or fountains, can encourage positive feelings and increase the frequency with which people access these areas [73].

In a study conducted in the city of Carmona, Italy, the authors found that the presence of playgrounds, pleasant views, drinking fountains, and recreational areas, all of which are linked to UGS quality, contributed to greater feelings of comfort and better perceptions of green spaces [74]. Likewise, in a nationwide study conducted in Norway by Fongar et al. (2019) [75], researchers observed that higher perceptions of quality positively influenced UGS visits and time spent there. In contexts where the understanding and quality of green spaces are similar, the pathway between UGS use and health through UGS quality may not be significant. However, in Latin American megacities where UGS quality exhibits high heterogeneity, this element can play a pivotal role, either enhancing or inhibiting the relationship [37].

Lastly, another critical pathway explored in this study links UGS use to health via time spent in UGS. Time spent in UGS also acts as a mediator between UGS quality and health and sentiments toward UGS and health, thus adding two additional pathways between UGS use and self-reported health. Despite some studies indicating that a higher frequency of UGS use is conducive to better health outcomes irrespective of the time people spend in UGS [76], evidence suggests that the extent of the benefits perceived is determined by the time that individuals spend in the green space [77–80]. For instance, it has been widely documented that the possibility of restoration in green spaces is expanded as the time spent there increases [81]. Further, time can influence the sentiments toward, or perceptions of a given space, affecting its use [33]. Consequently, the model introduced in this analysis includes time, as it is hypothesized that time partially mediates the relationship between UGS use and health.

## 2. Materials and methods

### 2.1. Materials

The study’s analytical approach relied on an online survey distributed through social media in May 2020. The decision to collect the data via this method was made because of the high risk of COVID-19 contagion through face-to-face data-gathering methods in 2020 [82]. For the survey design, ten in-depth interviews with experts on urbanism in Latin America were first conducted to identify critical questions and better understand the mediators influencing the pathways between urban green spaces and health in megacities of low- and middle-income countries. These semi-structured interviews took place in March and April 2020 via Zoom, each lasting between 60 and 90 minutes.

The final questionnaire was then uploaded to Qualtrics for two pilot tests, each involving five volunteers. Following the pilots, the survey was launched on Facebook in May 2020 using targeted advertisements directed at adults (18+) whose profiles indicated that they lived in one of the 16 municipalities of Mexico City. The participants’ location at the time of survey completion was verified through Qualtrics. Individuals outside the study area were excluded from the sample. Furthermore, the ad explicitly sought individuals who had been living in Mexico City for a minimum of 14 months. Facebook was selected as a distribution channel due to its status as the most popular social platform in Mexico, with over 81 million registered users [83]. The final questionnaire was translated into Spanish and included four sections: (i) UGS use, (ii) UGS quality and sentiments toward UGS, (iii) self-rated health, and (iv) sociodemographic characteristics.

For the purposes of this analysis, only surveys in which respondents completed all the questions were considered (n = 1,707, 51% completion rate). In order to encourage a higher level of participation, more detailed information like respondents’ specific income was not requested. Instead, respondents were prompted to select their income bracket. This approach aligns with standard practices in online data collection, acknowledging the common reluctance of individuals to provide precise personal information [84]. Additionally, this procedure was incorporated due to the documented mistrust that Mexicans have toward institutions and online data collection methods [85]. The ethical standards of this study were approved by the University of Melbourne’s Psychology Health and Applied Sciences Human Ethics Sub-Committee (2056618) on May 8, 2020.

### 2.2. Methods

Structural Equation Modeling (SEM) was chosen as the primary analytical method due to its capability to accommodate multiple dependent variables and account for mediating effects among interrelated variables [30, 86]. This approach has emerged as a quasi-standard in research, particularly for unraveling complex relationships among multiple variables and discerning both their direct and indirect impacts. In studies characterized by intricate research questions, where numerous factors influence outcomes, SEM proves exceptionally valuable. Notably, all variables incorporated in this model are manifest variables, and there are no latent variables present. In the following section, a detailed overview of the variables integrated into the model is shown and a graphical conceptualization of the study is presented for better clarity.

#### 2.2.1.Variables

*2*.*2*.*1*.*1*. *UGS use*. To assess UGS use and reduce the effect of the COVID-19 pandemic restrictions on respondents’ answers (enforced in Mexico City from March 23, 2020, to September 2021), participants were requested to select the option that most accurately represented their usage patterns of green spaces between March 2019 and March 2020 (spanning 12 months). The survey clarified that "use" specifically referred to direct engagement with these spaces. In other words, respondents were asked about their specific intention to visit green spaces, ensuring that they were not merely passing through them while traveling to another destination. Responses were classified as (i) no use (none), (ii) once every two-three months (rare), (iii) once or twice per month (occasional), and (iv) once or more than once a week (frequent). The survey differentiated the frequency of use based on prior research indicating that frequent use of UGS rather than sporadic use is significantly associated with improved health outcomes [81]. For example, a study conducted in the German city of Maihem highlighted that mere exposure to UGS is insufficient to benefit from them; consistent and regular use is necessary [87].

*2*.*2*.*1*.*2*. *Health*. To assess health outcomes, participants were asked to assess their perceived health status utilizing a 5-point Likert scale (1 = poor; 5 = excellent). This measure of self-reported health was employed as a proxy for health status, aligning with the approach outlined by Jylhä (2009) [88]. Jylhä’s research comprehensively discusses the advantages and limitations of self-rated health as a measure of health. It emphasizes that self-rated health stands as a valid measure of health status. Furthermore, several additional studies have validated self-reported health as a measure of health status, highlighting its predictive power for various health outcomes such as subsequent medical care utilization and mortality [89, 90].

In Mexico, researchers frequently use self-perceived health as a well-established proxy to study overall health. For example, Valle (2009) [91] investigated the relationship between social inequality and health, employing self-assessed health and reported morbidity as proxies for overall health. Similarly, in examining the factors contributing to poor health in Mexico, Gallegos-Carrillo et al. (2006) [92] used self-perception of health status as the main dependent variable. Moreover, a study by Flores et al. (2015) [93] in Cuernavaca, Mexico, revealed that self-perceived health serves as a reliable predictor of heightened cardiometabolic risk, an objective health indicator commonly used to establish patients’ health.

*2*.*2*.*1*.*3*. *Mediators*. As previously highlighted, this study incorporated three essential mediators: sentiments toward UGS, UGS quality, and time spent in UGS. To assess sentiments toward UGS, participants were requested to evaluate the significance of UGS to their overall quality of life and urban experience using a 4-point Likert scale (1 = not important at all; 4 = extremely important). Understanding individuals’ sentiments toward spaces is crucial, as this factor significantly influences their inclination to utilize these spaces, subsequently shaping their actual behavior [31]. Likewise, UGS quality was measured using a 5-point Likert scale, wherein respondents assessed the quality of green spaces in their neighborhood (1 = poor; 5 = excellent). Past research on Latin American cities, including Mexico City, suggests a strong correlation between UGS quality and improved health outcomes, as residents tend to favor clean, well-maintained green spaces abundant in vegetation, playgrounds, or sports facilities [94, 95]. Lastly, to define time spent in UGS, respondents were inquired about the average duration they typically spend during each visit to a green space. This variable was defined as a categorical variable in which the groupings included (i) 0 to 15 minutes, (ii) 16 to 30 minutes, (iii) 31 to 60 minutes, and (iv) more than 60 minutes.

*2*.*2*.*1*.*4*. *Additional exogenous variables*. Two fundamental sociodemographic attributes at the individual level were considered as exogenous variables in the model: age and socioeconomic status. It is well established that an individual’s health and well-being are significantly impacted by their age and socioeconomic circumstances [96, 97]. For instance, research conducted in Australia, focusing on health needs and beliefs, highlighted the association between age and health-related behaviors, emphasizing that a higher percentage of older individuals engage in health prevention strategies and undergo health checks [98]. Similarly, an online survey in Spain, exploring the effects of the COVID-19 pandemic on health, demonstrated that older individuals exhibited better mental health outcomes, characterized by lower levels of stress and anxiety compared to their younger counterparts during the turmoil [99] (Gonzalez-Sanguino et al., 2020).The work by House et al. (1990) [100] delved into the interconnection among age, socioeconomic status, and health, ultimately affirming that age and socioeconomic status are pivotal determinants of health outcomes. Respondents were asked to provide their age and socioeconomic status within defined ranges. Socioeconomic status was categorized into quintiles as per the definition by Mexico’s National Council of Evaluation (CONEVAL). Concurrently, age was grouped into six categories: (i) 18–25, (ii) 25–34, (iii) 35–44, (iv) 45–54, (v) 55–64, and (vi) 65 or more.

#### 2.2.2. Pathways in the SEM

**Fig 1:**
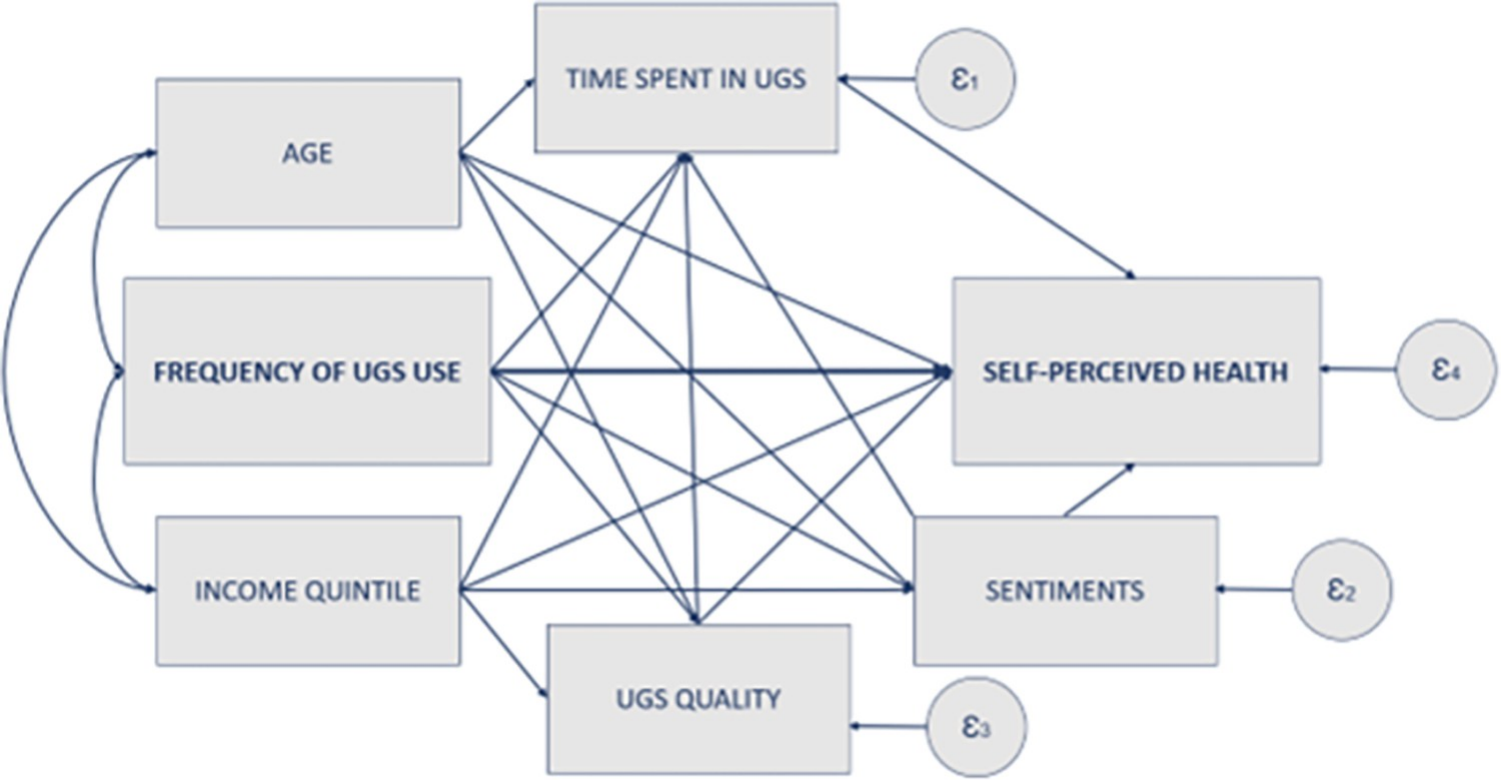
Conceptual model of the pathways between UGS use and perceived health.

The Structural Equation Model, illustrated in Fig 1, serves as the foundation for the subsequent analysis. It draws upon a body of literature delving into the intricate relationship between green spaces use and perceived health, employing solely manifest variables [31, 101]. Three primary mediators were accounted for in the analysis, namely sentiments toward UGS, UGS quality, and time spent in UGS. Furthermore, the analysis investigates two additional mediating routes within the relationship between UGS use and perceived health, including those where the time spent in UGS is mediated by UGS quality or the sentiments toward UGS. Consequently, the SEM reveals and elucidates five distinct indirect pathways connecting UGS use to perceived health:

Path 1: UGS use -> sentiments toward UGS -> self-reported health

Path 2: UGS use -> UGS quality -> self-reported health

Path 3: UGS use -> time spent in UGS -> self-reported health

Path 4: UGS use -> sentiments toward UGS -> time spent in UGS -> self-reported health

Path 5: UGS use -> UGS quality -> time spent in UGS -> self-reported health

When examining the pathways, several sociodemographic attributes were initially considered as exogenous factors or controls. However, after rigorous analysis, it was found that only age and socioeconomic status bore significant results. Consequently, these two variables were retained and integrated into the final model.

## 3. Results

Summary statistics of the study population are presented in Table 1. The data indicates a diverse range of respondents in terms of age, with the highest percentage (24.4%) falling into the youngest age group (18–24 years). This age distribution aligns with the 2020 Population and Housing Census in Mexico, highlighting the prevalence of this age group among legal adults in Mexico City [102]. Furthermore, the survey encompassed individuals from all income quintiles. The income quintile with the highest representation reflects the economic landscape of Mexico City, known for its highest per capita income in the country. Notably, a significant majority of respondents (69%) reported frequent use of UGS, underscoring their substantial utilization. However, only 13% rated their health as excellent, while the majority (35%) positioned their health in the middle of the scale (good).

**Table 1:** Summary statistics of the study population (n = 1,707, *online survey*, *May 2020*).

	n	%
**Age**		
18–24	417	24.4%
25–34	345	20.2%
35–44	250	14.6%
45–54	285	16.7%
55–64	251	14.7%
65 or more	159	9.3%
**Income quintile**		
1	295	17.3%
2	276	16.2%
3	252	14.8%
4	282	16.5%
5	602	35.3%
**Frequency of UGS use**		
none	26	1.5%
rare	122	7.1%
occasional	385	22.6%
frequent	1,174	68.8%
**Self-reported health**		
poor	225	13.2%
fair	326	19.1%
good	600	35.1%
very good	334	19.6%
excellent	222	13.0%
**Sentiments toward UGS**		
not important at all	121	7.1%
slightly important	223	13.1%
very important	240	14.1%
extremely important	1,123	65.8%
**Quality of UGS**		
poor	478	28.0%
fair	296	17.3%
good	409	24.0%
very good	312	18.3%
excellent	212	12.4%
Average time spent in UGS per visit*		
no use	26	1.5%
0–15 min	144	8.4%
16–30 min	533	31.2%
31–60 min	651	38.1%
more than 60 min	353	20.7%

* For the average time spent per visit in UGS, respondents were asked to choose the option that best described their patterns of use of green spaces from March 2019 to March 2020 (12 months).

In terms of sentiments toward UGS, a significant portion of respondents (66%) emphasized the paramount importance of UGS for both city life and their overall quality of life. However, a notable percentage held contrasting views: 7.1% regarded UGS as unimportant, and 13% considered them only slightly important. This finding underscores that approximately one-fifth of the surveyed individuals did not perceive green spaces as crucial to urban quality of life. Concerning the quality of green spaces in people’s neighborhoods, most individuals felt that this was poor (28%). Intriguingly, the highest rating—excellent quality—garnered the fewest responses, accounting for merely 12% of the participants. This disparity in perceptions sheds light on varying assessments of the local green space quality. Regarding the duration of visits to UGS, a significant majority (69%) reported spending between 16 and 60 minutes per visit. Remarkably, one-fifth of respondents allocated more than 60 minutes during each visit, highlighting the substantial investment of time in utilizing these green spaces.

### 3.1. Structural equation modeling

**Fig 2:**
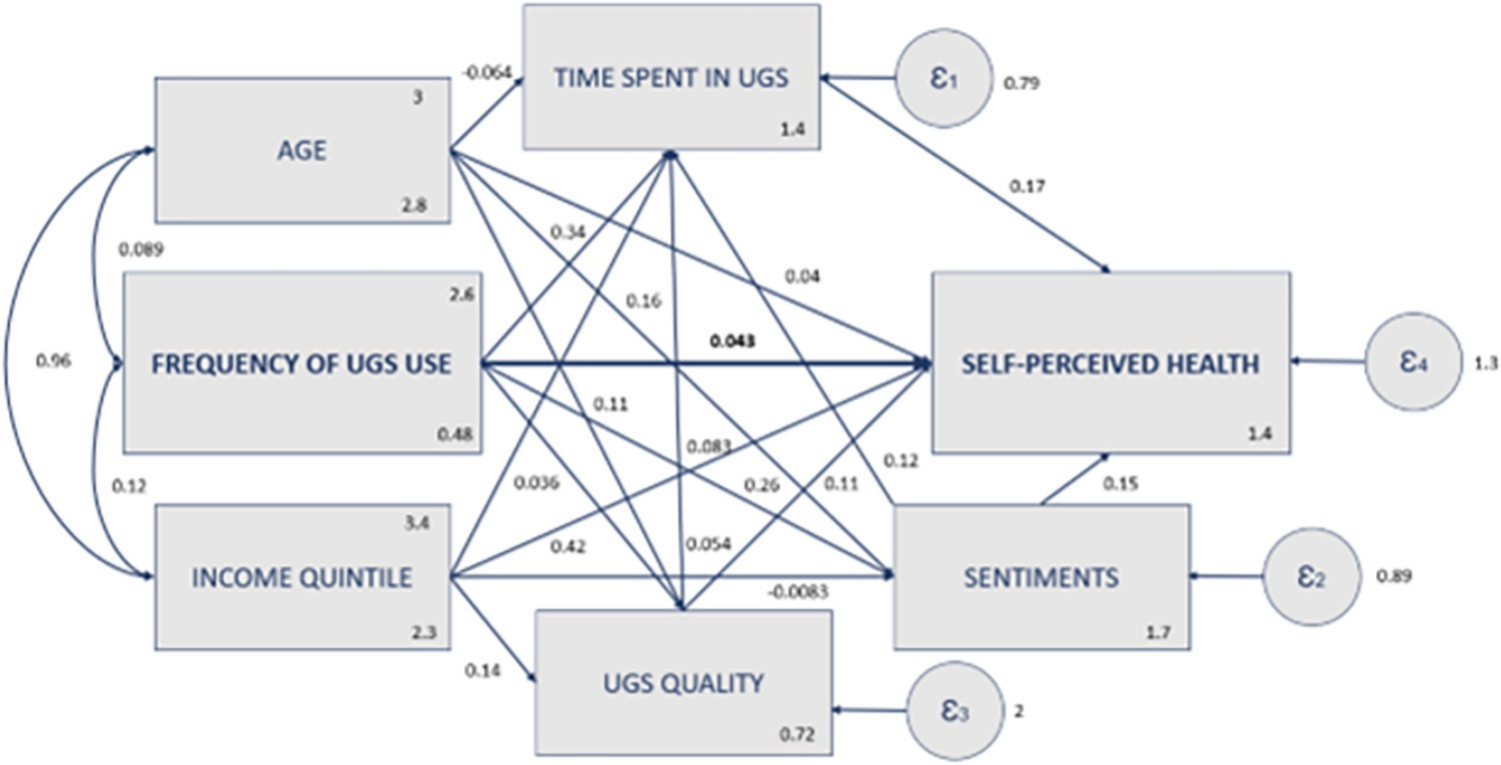
Final SEM model describing the direct and indirect effects of UGS use on self-rated health.

Fig 2 shows the final SEM model, delineating the direct and indirect pathways between UGS use and self-perceived health. A comprehensive evaluation of the model’s goodness-of-fit was performed using various indicators. The chi-square goodness-of-fit test yielded a *p*-value of 0.261. Additionally, crucial fit indices were scrutinized, namely the root mean square error of approximation (RMSEA = 0.012), the comparative fit index (CFI = 1.000), the Tucker-Lewis index (TLI = 0.992), and the standardized root mean squared residual (SRMR = 0.005). All of these metrics affirmed that the model exhibited a strong and favorable fit.

To test the total indirect effects of UGS use on perceived health, a bootstrapping method was applied. Many scholars recommend the bootstrapping method over a normal theory approach when testing indirect effects due to its robustness and accuracy [103]. This method has demonstrated effectiveness in estimating both standardized and unstandardized indirect effects. It involves resampling the raw data and generating an empirical distribution of indirect effect point estimates, allowing for the establishment of a confidence interval [104]. In this study, 2,000 bootstrap samples were utilized to construct percentile confidence intervals.

The results obtained through the bootstrapping method indicated a weakly significant direct path from UGS use to self-perceived health (coeff = 0.0427, *p*<0.10). Notably, the five indirect pathways explored in the model were highly significant (coeff = 0.1950, *p*<0.010), as outlined in Table 2. These findings provide substantial evidence that sentiments toward UGS, UGS quality, and the time spent during UGS visits significantly mediate the relationship between UGS use and health.

**Table 2:** Standardized direct, indirect, and total effects of green space use on self-reported health.

Pathways	Direct effects		Indirect effects		Total effects		C.I.
Path 1			0.0386	***			(0.0206–0.0593)
Path 2			0.0472	***			(0.0288–0.0690)
Path 3			0.0572	***			(0.0332–0.0842)
Path 4			0.0054	***			(0.0027–0.0090)
Path 5			0.0039	***			(0.0016–0.0068)
UGS use -> health	0.0427	*	0.15232	***	0.1950	***	(0.1097–0.2802)

(*p < 0.10

**p < 0.05

***p < 0.01)

Among the introduced exogenous variables used as controls, age was found to be significantly associated with respondents’ perceived health both through UGS quality (coeff = 0.0126, *p* <0.01), as well as time spent in UGS (coeff = 0.0030, *p* < 0.01), as outlined in Table 3. Similarly, income was found to indirectly influence perceived health through the same mediating factors, exhibiting strong significance for UGS quality (coeff = 0.0153, *p* <0.01) and moderate significance for time spent in UGS (coeff = 0.0061, *p* <0.05). Interestingly, sentiments toward UGS were not identified as mediators between age or income and perceived health.

**Table 3:** Standardized direct, indirect, and total effect of additional exogenous variables on perceived health.

Pathways	Direct effects		Indirect effects		Total effects	
age->sentiments->health			0.0025			
age->quality->health			0.0126	***		
age->time->health			0.0030	***		
age->sentiments->time->health			0.0003			
age->quality->time->health			0.0004	***		
age->health	0.0400	**	0.0056	***	0.0455	***
income->sentiments->health			0.0024			
income->quality->health			0.0153	***		
income->time->health			0.0061	**		
income->sentiments->time->health			-0.0002			
income->quality->time->health			0.0013	***		
income->health	0.0832	***	0.0213	***	0.1045	***

(*p < 0.10

**p < 0.05

***p < 0.01)

In terms of direct effects, income demonstrated a robust association with better-perceived health (coeff = 0.0832, *p* <0.01), albeit with a modest effect. Conversely, the direct effect of age on health was moderately significant (coeff = 0.040, *p* <0.05). Both age (coeff = 0.0455, *p* <0.01) and income (coeff = 0.1045, *p* <0.01) exhibited more substantial direct effects on self-reported health than UGS use (coeff = 0.0427, *p* <0.10), with their total effects being highly significant.

## 4. Discussion

The findings of this study underscore the significant but modest indirect effect of mediating factors—namely, sentiments toward UGS, UGS quality, and time spent in UGS—interceding in the pathway between the frequency of UGS use and self-reported health of residents of Mexico City. In terms of sentiments toward UGS, these results resonate with studies from the United States, Canada, and England, suggesting that an individual’s inclination to rate spaces favorably or unfavorably (the sentiments toward the space) significantly influences the degree to which the space’s use is associated with health outcomes [70, 105, 106].

In addition, this study unveils that the time spent in UGS acts as a mediator between sentiments toward UGS and health. In other words, spending more time in UGS is associated with individuals’ positive sentiments toward them. This finding aligns with the results of a study conducted in Turkey by Akpinar (2016) [80], which established that increased time in green spaces led to favorable perceptions of the spaces, including their features, which were associated with improved physical health outcomes. Similarly, a study conducted in Oslo, Norway, during the COVID-19 pandemic indicated that individuals with a favorable attitude towards green spaces tended to spend increased durations in these areas, resulting in enhanced well-being [107].

In order to enhance sentiments toward UGS, public health campaigns to promote a positive perception of UGS and reinforcing their importance for urban quality of life could prove to be a significant contributor to intensifying the relationship between UGS use and perceived [108, 109]. For instance, in Australia, Sugiyama et al. (2018) [110] have shown that public campaigns to modify existing sentiments can encourage healthier behaviors. Moreover, a systematic literature review by Hunter et al. (2015) [111] on the use of UGS, highlighted how interventions like the introduction of physical activity programs in urban parks can incentivize the use of these spaces, positively altering the perception or sentiment toward parks, leading to improved health outcomes. Beyond physical activity programs, apps and public messaging can also become effective inducements to modify sentiments or attitudes [112]. For example, in George County, Mississippi, the Department of Parks and Recreation introduced a tool to encourage and track park and trail usage, highlighting the importance of these spaces in improving people’s well-being, which yielded positive results for UGS use [113].

The SEM findings also suggest that the quality of UGS acts as a significant mediator in the pathway between UGS use and self-perceived health, with a higher perceived quality of UGS strengthening this relationship. Veitch et al. (2012) [114], who studied park use in Victoria, Australia, found that a sense of enhancement in the aesthetics—essentially, an improvement in the quality of the space—substantially boosted park use. Similarly, in a photo-journal study conducted in Mexico City, researchers found that residents who perceived the UGS in their neighborhood as high quality were more inclined to use them, with some residents even investing their own resources to improve the green spaces in their area [115]. The latter underscores the possibility to engage residents in the design, upgrading, or preservation of environmental settings to tentatively enhance the utilization and public perception of UGS. The engagement process could encompass employing tools such as surveys, checklists, meetings, focus groups, or consultations to discern the preferred types of facilities, activities, or operating hours for the relevant space [116].

Building upon analogous studies that employed quality as a mediating factor between UGS use and health [117], it is important to note that the survey utilized for this analysis did not precisely define the concept of "quality" concerning UGS. In this regard, attaining a more comprehensive understanding of the specific attributes constituting quality can inform more insightful analyses [115, 118]. Therefore, conducting additional research on the features, amenities, or activities that align with the community’s preferences and needs, thus defining the quality of UGS, is essential to understand how to strengthen the association between UGS use and health. In countries like Canada, Italy, Australia, and Indonesia, methods for defining and evaluating UGS quality, like community participation processes, have been integrated into park management to incorporate community needs into the planning and development of these spaces [119–121].

Buono et al. (2012) [122] have highlighted the necessity of establishing quality standards to ensure the effectiveness of green space upgrading practices. Therefore, creating a guide or setting essential principles to define the quality of UGS in Mexico City would be valuable. This approach would enable the inclusion of objective measures in the analysis, complementing the subjective perceptions of the space [118]. Moreover, having a clearly defined concept of quality might be beneficial for understanding the intrinsic connection between quality and safety. Several studies propose that, especially for women, quality and safety are intertwined and inseparable [123, 124], particularly in the Latin American in context [55, 125].

Finally, the findings also suggest that time spent in UGS acts as an additional mediator between UGS use and self-rated health. While some researchers have argued that visiting green spaces is sufficient to benefit from them, others have claimed that spending a certain amount of time in UGS is essential to reaping their benefits [126–128]. For instance, a study conducted in four European cities (Barcelona, Doetinchem, Kaunas, Stoke-on-Trent) by van den Berg et al. (2016) [129], which investigated the association between the time spent visiting green spaces and mental health using multilevel analyses, revealed a positive and significant association. Furthermore, a comprehensive meta-analysis encompassing 103 observational and 40 interventional studies found evidence to support the argument that individuals who spend more time being in green spaces experience significantly diminished risks of various chronic illnesses [4].

The SEM used in this analysis also revealed that the time spent in UGS serves as a mediating pathway between sentiments toward UGS or UGS quality and self-reported health. These findings align with the existing literature, indicating that the quality of UGS is linked to the time individuals spend in these spaces, consequently impacting the health outcomes of the users [130]. Similarly, these results align with recent studies in the UK, indicating that as the sentiments toward UGS became more positive due to the changes brought about by COVID-19, individuals expressed an increased eagerness to spend more time in these spaces, subsequently reporting positive health benefits [128, 131].

In this vein, introducing activities that encourage people to spend more time in UGS could prove to be an effective strategy to increase both the frequency and duration of UGS visits [132]. This, in turn, could enhance the positive association between UGS use and perceived health. Given the extensive evidence highlighting parks as ideal spaces for social interactions, integrating activities and programs in parks could be an effective approach to potentially extend the time people spend in UGS during each visit [133–135]. Nevertheless, further studies analyzing the mechanisms that foster increased interest and time spent in UGS within the context of Mexico are necessary for a comprehensive understanding of how to enhance this aspect.

## 5. Strengths and limitations

Although UGS research has gained traction in recent decades, substantial knowledge gaps persist. Particularly in Latin America, research in this domain has been [33, 37]. This study applied Structural Equation Modeling to explore various mediating pathways between UGS use and self-perceived health within one of Latin America’s megacities, Mexico City. Consequently, it stands as one of the few studies in this region delving into intervening factors affecting the relationship between green spaces and health. The findings of this study offer valuable insights to the government of Mexico City, suggesting strategies to strengthen the relationship between of UGS use and health in the city. These strategies include enhancing positive sentiments and attitudes towards these spaces, improving UGS quality, and introducing initiatives to encourage increased time spent in these areas. Studies akin to the current one play a crucial role in advancing our comprehension of the mechanisms linking UGS use and health in this region.

In terms of limitations, it’s vital to stress that SEM, while providing opportunities to examine direct and indirect effects, does not imply causality. Accordingly, causal relationships between variables cannot be inferred from the analyses conducted. Nonetheless, while this study is cross-sectional in nature, it is crucial to emphasize that the findings open intriguing avenues for future research, particularly in terms of UGS quality and sentiments toward green spaces. In addition, the model delves into individual-level variations, acknowledging that external factors, such as high crime rates, could inhibit the utilization of green spaces. Given the structural equation modeling framework of this study, exogenous factors were not specifically investigated.

Moreover, it is important to note that certain latent variables that have been shown to significantly mediate the relationship between UGS use and health in diverse studies, such as social cohesion [30], were not included in this study’s SEM. For instance, in a study conducted in 124 neighborhoods across four European cities, social cohesion was identified as a mediator for the frequency of green space visits and health [79]. Similarly, in a study on Dutch cities, researchers found that the positive health effects of greenery may be amplified by social cohesion [136]. Another study conducted by Cradock et al. (2009) [137] demonstrated that increases in physical activity in parks in Chicago, U.S., were associated with high levels of social cohesion. Nevertheless, social cohesion is not strictly an observable variable, and existing instruments or indicators to measure social cohesion have not been tested in the Latin American context, with definitions varying considerably [138].

Another limitation was the use of an online survey, which might have constrained the engagement of marginalized groups. Research has demonstrated that data collection tools relying on the Internet can inadvertently discriminate against underprivileged populations, as individuals without access to the internet at home are automatically excluded [139]. Nevertheless, given the prevailing risks and restrictions during the data collection period of this study (May 2020), an online survey was considered the least risky and most efficient method to gather insights into participants’ UGS usage patterns and their perceptions of green spaces. In addition, this data collection approach might have disproportionately excluded older adults due to potential challenges with technology, as they are typically less technologically adept, resulting in their underrepresentation among the respondents. Nonetheless, the survey managed to gather responses from all age groups. Lastly, there might be a selection bias towards individuals highly interested in the subject of UGS. This bias could also manifest in other data collection methods, such as phone surveys or mail questionnaires, as those less engaged with the subject might choose not to participate.

## 6. Conclusions

This study provided valuable insights into the relationship between the use of urban green spaces and the self-reported health among residents of Mexico City. Employing Structural Equation Modeling, the research effectively addressed notable gaps in existing literature by analyzing the multifaceted connections between UGS use and health outcomes, considering three critical mediators: sentiments toward UGS, UGS quality, and time spent in UGS.

The findings shed light on the nuanced relationship between UGS use and perceived health. They emphasize the importance of considering mediating factors to understand the intricate mechanisms through which UGS use influences self-perceived outcomes. While UGS use exhibited a weak and minor direct impact on self-reported health, the indirect pathways were notably robust, collectively bearing more substantive weight. Thus, in Mexico City, enhancing aspects such as quality of UGS, time spent in UGS, and sentiments toward UGS can potentially bolster the positive association between UGS use and self-reported health.

Furthermore, the study underscored the need for targeted interventions and thoughtful policy formulation to cultivate favorable perceptions of UGS, elevate UGS quality, and encourage extended periods spent in these spaces. Public health campaigns, engagement initiatives, and activity programs within UGS might be effective tools to achieve these objectives and enhance the public health outcomes associated with UGS use. Nonetheless, additional research on these instruments is needed.

Finally, this study is important in its contribution to the discussion on the impact of nature-based solutions on public health in Latin America, where studies are still scarce. As research in this field continues to grow, more evidence is expected to emerge regarding the association between UGS use on health and well-being in Latin American urban settings, and the pathways to strengthen this association. Researchers, policymakers, and urban planners are increasingly recognizing the importance of green spaces in creating healthier and more livable cities in the region. In this regard, this study provides a solid foundation for future studies and policy interventions aimed at optimizing the health benefits derived from UGS and enhancing the overall livability of urban environments, not only in Mexico City but also in similar settings globally.
